# Hydrothermal Liquefaction: How the Holistic Approach by Nature Will Help Solve the Environmental Conundrum

**DOI:** 10.3390/molecules28248127

**Published:** 2023-12-16

**Authors:** Saeed Ranjbar, Francisco Xavier Malcata

**Affiliations:** 1 LEPABE—Laboratory for Process Engineering, Environment, Biotechnology and Energy, Faculty of Engineering, University of Porto, Rua Dr. Roberto Frias, 4200-465 Porto, Portugal; up202000001@edu.fe.up.pt; 2 ALiCE—Associated Laboratory in Chemical Engineering, Faculty of Engineering, University of Porto, Rua Dr. Roberto Frias, 4200-465 Porto, Portugal

**Keywords:** hydrothermal liquefaction, biomass, waste, sustainability, energy

## Abstract

Hydrothermal liquefaction (HTL) represents a beacon of scientific innovation, which unlocks nature’s alchemical wonders while reshaping the waste-to-energy platform. This transformative technology offers sustainable solutions for converting a variety of waste materials to valuable energy products and chemicals—thus addressing environmental concerns, inefficiencies, and high costs associated with conventional waste-management practices. By operating under high temperature and pressure conditions, HTL efficiently reduces waste volume, mitigates harmful pollutant release, and extracts valuable energy from organic waste materials. This comprehensive review delves into the intricacies of the HTL process and explores its applications. Key process parameters, diverse feedstocks, various reactor designs, and recent advancements in HTL technology are thoroughly discussed. Diverse applications of HTL products are examined, and their economic viability toward integration in the market is assessed. Knowledge gaps and opportunities for further exploration are accordingly identified, with a focus on optimizing and scaling up the HTL process for commercial applications. In conclusion, HTL holds great promise as a sustainable technology for waste management, chemical synthesis, and energy production, thus making a significant contribution to a more sustainable future. Its potential to foster a circular economy and its versatility in producing valuable products underscore its transformative role in shaping a more sustainable world.

## 1. Introduction

In the vast expanse of our shared planet, critical challenges stemming from waste generation and management demand immediate attention and pursuit of sustainable solutions. Alarming statistics forecast a 70% increase in global waste generation, projected to reach 3.4 billion tons annually by 2050. This impending crossroads results from the convergence of population growth, urbanization, and industrialization, leading to unsustainable consumption patterns. One poignant chapter in this narrative is food waste, with ca. 1.3 billion tons of edible waste generated annually, contributing to the release of 3.5 billion tons of carbon dioxide into the atmosphere. Across various regions, from Europe to America and from Africa to Asia, staggering amounts of food end up as waste, underscoring the urgent need for effective waste-management strategies. Sewage sludge, a challenging byproduct of municipal wastewater treatment, has traditionally been managed using methods with limited efficiency, thus posing contamination risks and resulting in high costs. To tackle these challenges, the principles of circular economy have been suggested as a way to address them. Coupled with increasing public awareness, such principles are driving society to adopt innovative approaches and effective policies [1,2,3,4,5,6].

Conventional waste biomass conversion technologies fall into two main categories: thermochemical and biological processes. Thermochemical methods, like liquefaction, pyrolysis, gasification, torrefaction, carbonization, transesterification, and combustion, rely on high temperatures or chemical catalysts to break down biomass. These processes often yield valuable products, such as biofuels, syngas, and biochar, but they typically demand substantial energy inputs and may produce complex mixtures requiring extensive refining afterward. In contrast, biological processes, like fermentation, biomethanation, and enzymatic reactions, operate in milder conditions, using microorganisms or enzymes to convert biomass into fuels, chemicals, or gases. Although they have lower energy requirements and broader feedstock flexibility, they are slower and need accurate environmental control, thus potentially limiting scalability and consistency [7].

Within this context, hydrothermal processing emerges as a transformative technology—encompassing hydrothermal carbonization (HTC), hydrothermal liquefaction (HTL), and hydrothermal gasification (HTG). At its core, HTL invokes a water-driven thermochemical marvel—releasing large organic molecules as highly active smaller counterparts within an enclosed reactor devoid of oxygen. HTL bestows upon us a magnificent tapestry of products, including biocrude oil, water phase, solid residue, and gases. Biocrude oil, with an average higher heating value (HHV) of 30 MJ/kg, can be further transformed into liquid fuels through catalytic hydrogenation or distillation. The water phase offers the potential for recirculation and reuse or serves as a medium for microorganisms within bioreactors. The solid residue, biochar, entails valuable applications in soil amendment, water treatment, and even as a solid fuel or precursor for nanocarbon materials. Lastly, the gas phase offers diverse opportunities as fuel, namely hydrogen production and fermentation by gas-fermenting microorganisms [8,9,10,11,12,13,14,15,16].

## 2. Hydrothermal Liquefaction Process 

### 2.1. Principles and Reaction Pathways

**Figure 1:**
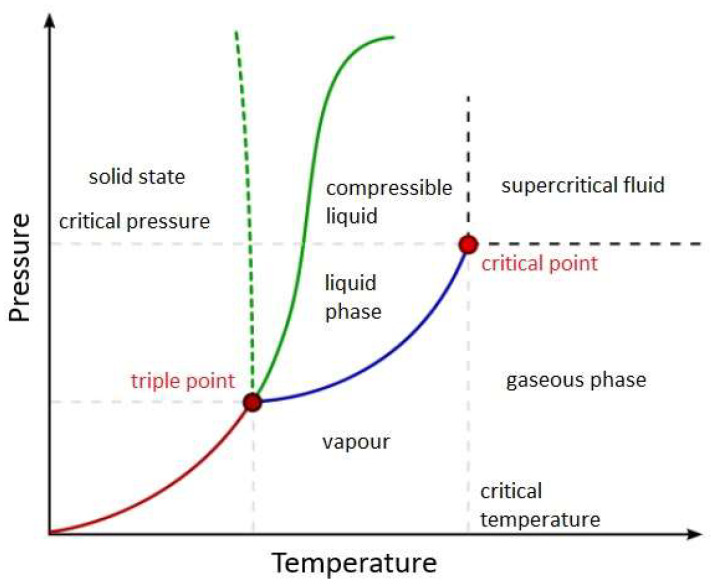
Phase diagram of water, depicting changes in physicochemical properties with temperature and pressure.

In the realm of HTL, a remarkable metamorphosis unfolds—with water as the central player, wielding a pivotal role in transformative processes amid heightened temperatures. Within this aqueous milieu, subcritical water exhibits exceptional characteristics—showcasing elevated ionization constants and yielding a profusion of ionic products, notably H_3_O^+^ and OH^−^. These dynamic entities possess the unique ability to dismantle complex macromolecules and deconstruct them into their fundamental building blocks. This process sets the stage for the subsequent reassembly, thus enabling the generation of a diverse array of HTL-derived products. Under high temperature and high pressure, water reveals its multifaceted nature in liquid and gaseous states (Figure 1). Beyond its critical point, water seamlessly navigates without undergoing phase transitions and thus enters the fabled supercritical state. In this form, water assumes a dual role—acting both as a reactant and a catalyst and exerting a remarkable influence on the HTL stage. 

**Figure 2:**
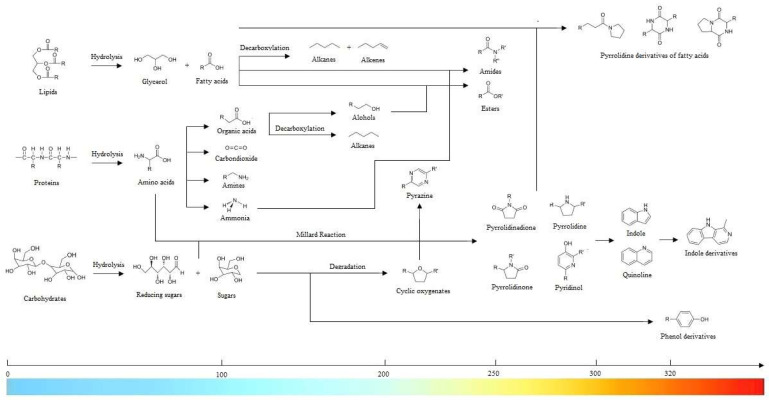
Chief HTL-mediated reaction pathways [22].

In the supercritical state, water proves to be an exceptional solvent for most homogeneous organic reactions—endowed with high miscibility and thus free from phase boundaries. Such extraordinary attributes engender higher reaction rates, where nucleophilic substitutions and eliminations flourish. Moreover, water sheds its viscosity as temperature rises, thus paving the way for elevated diffusion coefficients and mass transfer rates that are also nuclear in the transformative processes. A momentous drop in ionic products at the supercritical point fosters heterolytic cleavage of aromatic compounds and catalyzes acid/base reactions; a brief overview is conveyed in Figure 2. Accordingly, the delocalization of π-electrons, brought about by the substitution of hydroxyl groups, infuses the atmosphere with an air of instability, which accelerates free radical reactions and opens rings within heterocyclic compounds [17,18,19,20,21,22].

As temperature surpasses 210 °C, the microcrystalline structure of raw materials and the hydrogen bonds between polymeric chains dissolve, leading to noteworthy synergisms. Components like cellulose and hemicellulose, prevalent in lignocellulosic biomass, undergo physicochemical transformations—yielding oligosaccharides, monosaccharides, and such other products as furfural, hydroxymethylfurfural, and acetic acid. Amid this grand orchestration, sugars like glucose, xylose, xylan, arabinose, mannose, and galactose embark on individual journeys, thus engaging in isomerization, cyclization, dehydration, and condensation. These sugars merge harmoniously with phenols, ketones, uronics, acetaldehyde, glyceraldehyde, lactic acid, formic acid, acetic acid, and other low-molecular-weight acetyl groups, ushering forth a tapestry of exquisite final products. Furthermore, nitrogen-containing proteins gracefully surrender to hydrolysis, releasing a wide array of amino acids. These building blocks then embark on diverse pathways—undergoing decarboxylation to yield carbonic acid and amines or proceeding through deamination reactions, where large amounts of ammonia and organic acids are released. Finally, the encounter between reducing sugars and amino acids via Maillard reactions leads to the formation of melanoidin-like polymers and polycyclic compounds. Following this prelude, the melody continues as they decompose—and culminate in the emergence of such monocyclic compounds as pyrroles, pyrazines, indoles, and aromatic amines [17,18,19,20,21,22,23,24,25,26,27,28,29].

### 2.2. Key Process Parameters

The quality and quantity of products in the HTL process are greatly influenced by several key factors. These parameters encompass temperature, pressure, heating rate, preloaded pressure, residence time, feedstock characteristics, catalysts, solvent-to-feedstock ratio, particle size, and pH. 

Hydrothermal processes are generally endothermic at low temperatures but become exothermic at high temperatures. As a fundamental force, high temperature is essential to overcome the underlying energy barrier and use sufficient energy to activate biomass fragmentation toward achieving higher concentrations of free radicals during HTL. Typically, bio-oil yield increases with temperature up to a point where a further rise in temperature suppresses liquefaction and enters the gasification phase—with secondary decomposition and Bourdard gas reactions dominating and high concentrations of free radicals recombining into char. On the other hand, at temperatures below 275 °C, bio-oil yield also shows a decline due to the partial breakdown of biomass components; hence, a temperature range of 300–350 °C is considered necessary for greater bio-oil yields, and lower solid and gas production [30,31,32,33,34].

Marinating high pressure during HTL circumvents the energy costs of a two-phase system by retaining water in the liquid state. Under supercritical conditions, higher pressure results in higher local solvent density, which prevents C-C bond fragmentation. Therefore, an increase in pressure results at first in effective penetration and extraction of biomass, while it becomes insignificant at supercritical conditions and has little impact on bio-oil [30,35,36,37,38].

Residence time affects product composition and conversion efficiency of HTL. As time elapses, degradation under supercritical conditions proceeds rapidly and reaches its zenith. Short residence times favor maximum yield, while longer residence times result in the dominance of secondary and tertiary reactions. Heavy intermediates relinquish their place, forming a mixture of liquids and gases that subsequently diminishes bio-oil yield. Throughout the vast repository of HTL literature, reaction times on the order of tens of minutes have been reported, while a variation on the HTL process, termed fast HTL, has also been examined that requires just a short reaction time (up to about 2 min) and rapid heating rates (150–300 °C/min)—which yield high biocrude outputs while consuming comparatively lower energy. Such a process was tested with success on various feedstocks, including microalgae, macroalgae, bacteria, and yeast, to demonstrate its robustness [31,39,40,41,42]. 

As in other chemical reactions, feedstock composition and particle size play vital roles in hydrothermal processes. Hemicellulose and cellulose, with their amorphous structures and intermediate degrees of polymerization, are susceptible to degradation and thus significantly boost bio-oil yield. In contrast, the decomposition of lignin is limited by its high degree of polymerization and complex interlinkages, leading to residual fractions. Furthermore, HTL of loosely packed biomass results in bio-oil with high oxygen and moisture content and lowers the quality and HHV of the fuel. In terms of particle size for HTL, it has been reported that small particle size improves accessibility and penetration of heat, therefore improving conversion rate and bio-oil efficiency. However, it must be noted that excessive grinding results in increased processing costs [30,31,43,44,45,46].

The pH of the reaction environment in HTL can affect the formation of intermediates, the solubility of different components in the biomass, stability, and composition of the bio-oil produced, as well as catalyst activity thus significantly influencing the yield and quality of the final products. Optimal pH conditions depend on the type of biomass and the specific parameters of the HTL system at stake. In one instance, the effect of pH on the hydrothermal depolymerization of softwood kraft lignin was investigated. This was accomplished by adding KOH in a continuous small pilot unit with ZrO_2_ and K_2_CO_3_ as catalysts and phenol as a capping agent to suppress repolymerization. It was reported that the yield of water-soluble organics and bio-oil increased with pH, while the char yield on the zirconia catalyst showed a minimum at pH 8.1 but increased at higher pH values. At that pH, there was additionally a notable decrease in the yield of suspended solids and the oxygen content in the bio-oil, thus contributing to an overall enhancement in the quality and quantity of HTL products [38]. 

HTL marks a fascinating arena in our exploration of evolutionary pathways. Delving into this process unravels a profound realization echoing Darwin’s insights on adaptation, efficiency, and harmony within the confines of a high-temperature, high-pressure environment. Such conditions serve as the crucible that fosters the evolution of robust polymers and enduring chemicals. Over time, they metamorphose into smaller, more manageable, and biodegradable molecules, reclaiming a pathway toward integration within the living world. The essence of HTL lies in its transformative power, breaking down complex compounds into simpler, environmentally friendly constituents. This metamorphosis presents a promising opportunity to alleviate the burden of non-degradable materials on our planet. However, the intricacies of HTL feedstock pose challenges, as they may contain persistent elements resistant to facile breakdown. To overcome this hurdle, increased catalytic activity becomes imperative. Augmenting the catalytic process can enhance the efficiency of hydrolyzing these resilient elements by enabling their conversion into more easily manageable components. This catalytic intervention serves as a critical juncture, a fine-tuning of the HTL process to ensure the extraction of maximum value from the feedstock while minimizing environmental impact. The convergence of scientific inquiry with the principles of natural selection in HTL represents not just a technological advancement but a symbiosis with nature’s design, an intersection where human ingenuity aims to mimic and complement the inherent processes of nature, steering us toward a more sustainable coexistence with the environment senso latu.

### 2.3. Catalysts and Their Role in HTL

Catalysts assist biomass decomposition by decreasing the activation energy of chemical reactions in HTL. A grand cycle of reactions, encompassing ester formation, dehydration, deoxygenation, decarboxylation, and dehydrogenation, can be accelerated and optimized using homogeneous and heterogeneous catalysts. Homogeneously synthesized catalysts featuring alkali salts like Na_2_CO_3_, K_2_CO_3_, and KHCO_3_, as well as other catalytic forms like NaOH, KOH, and CO_2_, facilitate the water-gas shift reaction and mitigate char/tar formation. They offer the advantage of decreased solids production, increased biocrude yield, and improved biocrude properties. However, the challenge of costly separation—as they mix completely with reactants, poses a formidable hurdle downstream [47,48,49,50]. 

On the other hand, heterogeneous catalysts, including noble metals such as Pt, Ni, Pd, Ru, and various metal compounds like MnO, MgO, NiO, ZnO, CeO_2_, CuO, Al_2_O_3_, La_2_O_3_, and zeolite, showcase their own brilliance on the HTL stage. Among these, nanocatalysts composed of carbon-based materials, like carbon nanotubes, activated carbon, and graphene, exhibit unique surface characteristics and exceptional properties. Nanocatalysts address the limitations of both homogeneous and heterogeneous counterparts and provide potential solutions for catalytic HTL and biodiesel production. Carbon nanotubes demonstrate high stability under hydrothermal conditions and enhance bio-oil quality by increasing hydrogen content while reducing oxygen, nitrogen, and sulfur levels. Reduced graphene oxide (RGO) supports Ni catalysts, therefore enhancing the yield and quality of bio-oil produced from HTL of *Spirulina*. Functionalized graphene oxide/polyurethane composites shine as promising metal-free catalysts for upgrading biocrude derived from macroalgae. Waste-based nanocatalysts generate higher biodiesel yields compared to conventional homogeneous catalysts while maintaining their activity across multiple cycles—which introduces them as sustainable and efficient candidates [39,49,51,52,53,54,55,56,57]. 

The combination of heterogeneous and homogeneous catalysts invokes a synergistic effect on bio-oil production during HTL. This leads to improved deoxygenation and denitrogenation of bio-oil—with catalysts like K_2_CO_3_, ZrO_2_, CuO, and NaOH, or Ru/C and Raney Ni improving efficiency and yield [47,50,54,58,59,60,61]. In a visionary exploration, metallic nanoparticles on micron-sized bacterial cells emerge as an alternative to traditional homogeneous catalysts. These bio-catalysts, with environment-friendly and cost-effective support materials, grant the possibility of recycling and reusing metals from various waste sources—thus supporting an alluring avenue in a world of limited global supplies of such metals [62].

Co-solvents play a significant role in the ballet of HTL. Ethanol [63,64], methanol [65,66], toluene [67], phenol [37,48], isopropanol [68], formic acid [69], glycerol [70,71], propylene glycol [72], dichloromethane [40], and transition metal chlorides [73] act as scavengers of unsaturated molecules formed through dehydration, thus preventing their repolymerization. The synergy of water-alcohol mixtures enhances performance by reducing temperature and residence time requirements while introducing extreme non-polarity and increased acidity. As a result, lignin degradation is intensified, as prompted by enhanced diffusion and surface tension—although challenges in separation and technical aspects may temper their appeal for HTL [36]. Recent developments in click chemistry and nanotechnology promise an era of super-catalysts with improved efficacy. The graceful coordination of catalysts, driving selectivity and efficiency, ultimately holds the promise of a sustainable and harmonious future, in the realm of renewable energy and resource transformation [39,74]. Table 1 summarizes the main studies on catalytic hydrothermal liquefaction of biomass.

### 2.4. Biocrude Upgrade

Refining the products of HTL is a necessary step to prepare them for specific applications downstream. The gas and water fractions are often used with minor adjustments, while biochar can be upgraded to nanocarbon materials such as grapheme following acid treatment and dehydration. Biocrude is typically upgraded via catalytic hydrogenation. 

Countless studies have explored the hydrotreatment of biocrude from continuous HTL plants, revealing the impact of catalysts and distinct conditions on deoxygenation and yield. Crucial to achieving deoxygenation, such catalysts as Pt/Al_2_O_3_ and NiMo/Al_2_O_3_ exhibit promising results. Temperature and pressure also wield their influence upon deoxygenation, while the presence of nitrogen in the feedstock affects their performance to some degree. Biocrude hydrotreatment is carried out in batch autoclaves or continuous reactors. Within this interplay, continuous systems exhibit comparably higher yields of upgraded oil. Carbon yields exceeding 60% on a biomass basis, attained via combined processes, corroborate the effective removal of heteroatoms and saturation of hydrocarbons [54,61,62]. 

Beyond catalysts, other methods play a part in upgrading biocrude oil. Physical methods, such as distillation, and chemical methods, such as visbreaking and delayed coking, offer additional opportunities for upgrading. Fractional distillation, in particular, presents itself as a cost-efficient and energy-saving technique, leading to relevant optimization of resources. Unlike catalytic methods, this physical separation process offers possibilities to integrate biocrude into existing fuel supply chains. Extensive studies on the fractional distillation of lignocellulosic and algal feedstocks confirmed significant changes in biocrude composition [39,51,58,59,62,90,91,92,93,94,95,96,97,98].

## 3. Reactor Designs and Operation Modes 

Hydrothermal processing is characterized by an interplay of water, biomass, and catalysts within an autoclave. This process involves carefully controlled heating to specific temperatures, under high pressure, and for defined durations. The batch process offers such advantages as high dry matter content (20–30%) without concerns of pipeline obstruction or feedstock pressurization. However, shortcomings are apparent—e.g., thermal transience, difficulty in decoupling temperature and pressure effects, and challenges in scaling up to industrial production.

To date, in-depth analyses have focused on the challenges associated with HTL processes—concerning reactor construction, heat recovery, compound formation, and separation techniques. The demanding conditions—high critical water temperature and pressure, require substantial heat input and robust reactor materials. However, the high viscosity of the biomass creates poor heat transfer conditions, thus necessitating larger heat exchange areas and thicker walls for reactors and heat exchangers that impact heat recovery efficiency. Large-scale HTL reactors face challenges in raising reactant temperatures leading to carbonization, reducing biocrude yields, and demanding longer reaction times, larger reactors, and higher energy inputs. Moreover, emulsification during HTL complicates biocrude–solid separation, while the formation of corrosive compounds and high nitrogen levels in the biocrude pose material and emission concerns. Existing methods for nitrogen removal or biocrude upgrade often lack efficiency and cost-effectiveness, making the development of large-scale, economical HTL processes a complex endeavor. Upgrading methods, such as filtration, hydrotreating, blending, or steam reforming, present limitations in energy efficiency, catalyst usage, corrosiveness, or low biocrude production compared to fossil fuels. Despite these challenges, blending biocrude with fossil crude for conventional refining remains a viable option, provided that certain biocrude specifications are met [99]. 

Designing continuous HTL systems—much like composing a symphony, requires artful optimization of heat integration, graceful handling of high viscosities, minimalistic selection of reactor CAPEX, and harmonious choice of suitable materials, including pumping apparatuses. One of the primary issues arising from pumping a high-viscosity slurry is the viscosity changes that can occur with shear. This phenomenon, known as shear-thinning or shear-thickening, can cause changes in slurry viscosity as it moves through pumps and pipelines. When subjected to shear forces during pumping, some materials might decrease in viscosity, thus making them easier to pump. However, others might thicken, which would make pumping more challenging and require higher energy inputs. In HTL, where various organic materials are processed under high temperatures and pressures, the resulting slurry can exhibit diverse compositions, leading to non-Newtonian behaviors. These behaviors may be unpredictable and vary throughout the process, complicating the pumping process even further. Additionally, the presence of solids or particulate matter within the slurry can lead to abrasive wear on pumps and pipes, potentially reducing their lifespan and necessitating frequent maintenance. To address these challenges, engineers often explore specialized pump designs capable of handling high-viscosity and non-Newtonian fluids. Progress in pump technology and the development of systems designed for challenging rheological behaviors are prone to mitigate these issues to some extent. However, the complex nature of HTL-derived slurry demands continuous research and innovation to optimize pumping systems and reduce operational hurdles associated with viscosity changes and rheological complexities [100,101,102].

Continuous HTL systems provide better pressure and temperature control compared to batch reactors. Scaling up fuel production and overcoming high-pressure pumping challenges turn continuous HTL into an essential pursuit in this transformative technology. Further research critically hinges on reactor type and heating sources, as they significantly influence biocrude yield and economic feasibility. Tubular reactors, owing to their scalability and simplicity, as well as other innovative designs such as continuous stirred tank reactors (CSTRs), offer promising solutions for these issues. The motion of impellers for reactor agitation in CSTRs ensures proper mixing, while the hydrodynamic flow patterns—whether turbulent or laminar, influence the outcome [45,103,104,105,106,107].

Implementing efficient heat exchange mechanisms within the HTL system can substantially reduce energy demands and operational costs. For instance, employing heat exchangers to capture and reuse heat from the reactor effluent before its discharge could improve overall energy efficiency. Additionally, cascading heat from other processes within a plant or employing waste heat recovery systems could be explored to offset energy requirements. The heat required for HTL is typically supplied via external sources, such as electrical heaters or combustion systems. These systems resort to electricity or fossil fuels, thus contributing to the overall energy footprint of the process. Alternative heat sources could include renewable energy inputs such as solar, geothermal, or biomass-derived sources, aiming to reduce reliance on non-renewable resources. Concentrated solar power (CSP) appears as a captivating option—and offers energy efficiency and sustainability benefits. Recently, there has been an increasing interest in the use of microwave-assisted HTL. Upon microwave irradiation, the dipoles of water molecules align with the electric field of the microwave and rotate at high speed to generate heat; the carbon-rich biomass absorbs the energy and efficiently decomposes due to the rapid and uniform distribution of heat. This results in a general increase in the quality of HTL products and reduces the upgrading costs. Comparatively easier control of the process in terms of rapid initialization and termination, as well as decreased reaction time, are other attributes of microwave-assisted liquefaction, whereas high costs can be considered to be the main obstacle to be addressed in the future [108,109,110,111,112,113,114,115,116,117,118,119]. 

The pilot plant showcase of continuous HTL processes is characterized by rapidly increasing versatility in converting diverse biomass into valuable biocrude. The relentless pursuit of researchers and companies alike—tackling challenges, optimizing parameters, and refining the brilliance of this technology, has indeed propelled it forward as a nuclear player in the symphony of sustainable biofuel production from renewable biomass. In the evolutionary journey of biomass liquefaction, numerous institutions have actively innovated and played pivotal roles in advancing HTL technology (Table 2). As a result, several continuous pilot plants have been established across academic and industrial scales, refining HTL processes for diverse biomass types. Each contributes uniquely to the seamless production of sustainable biofuels, playing an instrumental role in developing proprietary technologies and demonstration plants. Their collective efforts have revolutionized the landscape of biocrude production [35,39,53,55,57,67,71,72,73,74,75,76,77,78,79,80,81,82,83,84,85,86,87,88,89,90,91,92,93,94,95,96,97].

## 4. Feedstock Selection and Preprocessing 

Biomass is a naturally abundant and widely distributed resource, surpassing traditional fossil fuels in its equitable distribution worldwide. Many materials have undergone the transformative process of HTL, but there is no single, definitive optimal solid-to-water ratio; instead, each feedstock requires accurate determination through empirical investigation. An ideal moisture content, ranging from 65% to 95%, facilitates pumpability and seamless slurry transportation to the HTL reactor. However, if this range is exceeded, larger reactor volumes are necessary, which entails increased capital and operating costs. Notably, particle size greatly influences HTL efficiency, with smaller solids supporting increased surface-to-volume ratios and elevated bio-oil yield. Due to their inherently smaller particle sizes, wet wastes like sludge and microalgae exhibit faster conversion rates and accordingly make the process economically viable [94,100]. 

Researchers have explored the application of HTL on waste feedstocks from various sources, including dairy industries (manure and yogurt whey), municipal wastewater treatment plants, dining halls, fruit and alcohol manufacture, and olive oil production. Other biomass sources, such as swine manure, watermelon peel, spent coffee grounds, crop straws, spent mushroom compost, and different types of wood, have also been investigated for HTL conversion. Different feedstocks produce varying bio-oil yields due to differences in their lipid, oxygen, nitrogen, sulfur, and ash contents—which, in turn, affect the resulting HHV. Microalgae, with high lipid content and low oxygen levels, typically yield 15–55% bio-oil with an HHV of 30–40 MJ/kg, comparable to petrodiesel fuel at around 46 MJ/kg. Conversely, macroalgae, primarily composed of protein, exhibit lower bio-oil yield and HHV. For instance, *Chlorella* yields 41.7 wt.% biocrude, while *Nannochloropsis salina* produces 54% bio-oil with an HHV of approximately 38.8 MJ/kg. Despite being promising, algal bio-oil raises a few challenges, such as high viscosity and nitrogen content, which necessitate further refinement. Hydrothermal processing of sewage sludge at 325 °C, with no holding time, yields ca. 26% bio-oil with an HHV of 27.7 MJ/kg. Furthermore, secondary pulp/paper sludge powder in HTL results in 20–45 wt.% water-soluble oils and 15–25 wt.% heavy oils, with HHVs of 10–15 MJ/kg and 435 MJ/kg, respectively. In another study, the conversion of human feces into biocrude oil, along with recovery of nutrients and metals, succeeded in the efficient degradation of waste without the need for pretreatment—and yielded up to 34.44% biocrude, with an HHV of 40.29 MJ/kg. Pilot plant experiments with waste streams have produced promising results, with biocrude yields ranging from approximately 48% to as high as 61% [6,18,19,20,24,27,33,34,40,44,49,60,61,121,123,126,130,133,150,151,152,153,154,155,156,157,158,159,160,161]. 

Continuous HTL systems have proven to be more robust and stable compared to batch processing by delivering consistent results even under changing reaction conditions. This field has seen innovative approaches, such as resorting to water phase recirculation and using organic and inorganic co-solvents to boost biocrude yield and enhance product quality. Co-liquefaction, where different feedstocks are combined, has shown clear advantages–resulting in synergistic effects that increase biocrude yield and improve its quality. Furthermore, mixing high-moisture feedstocks like algae or sewage sludge with drier feedstocks such as wood pellets or sawdust not only reduces water consumption but also eliminates the need for expensive dewatering steps. Introducing hydrogen-rich co-reactants, like plastic waste, during HTL enhances biocrude yield and offers a promising solution to waste-management challenges. Predictive models have also been developed to assess yield, thus helping identify optimal feedstock combinations and providing insights into the complex interactions involved in hydrothermal co-liquefaction. As the field of biotechnology continues to advance, HTL holds great promise in shaping a sustainable and efficient future for bioenergy production, waste management, and resource utilization [10,23,31,35,41,44,45,46,71,94,100,102,103,104,105,107,108,112,121,123,127,128,129,130,132,133,134,138,150,151,152,153,154,156,159,162,163,164,165,166,167,168].

## 5. Applications of Hydrothermal Liquefaction

The realization of the full potential of the biomass conversion process through HTL hinges on the effective valorization of its products, as outlined in Table 3. HTL of biomass results in five primary product streams: biochar, heavy bio-oil/chemicals, light bio-oil/chemicals, aqueous phase, and gas. It is imperative to implement efficient and economically feasible separation methods for these products to mitigate the environmental risks associated with their unprocessed release.

As a solid residue of HTL, biochar is considered a sustainable source for the production of carbon materials with adjustable surface properties, such as porous carbon, heteroatom-doped biochar, carbon nanotubes, graphene, and carbon quantum dots. These materials hold great promise for numerous applications, including semiconductors, supercapacitors, and construction materials with outstanding properties. Biochar and hydrochar are also frequently used as catalysts and fertilizers and for the bioremediation of wastewater and contaminated soil [53,89,160,172,173,186,187,188,189,190,198,199,200,201,202].

The aqueous phase (AP) produced through HTL offers promise for various applications, particularly as fertilizer, due to its richness in essential nutrients. It has been reported that, depending on the actual reaction conditions, nearly one third of the organic carbon in the feedstock and most nitrogen-containing compounds—produced by deamination of amino acids toward generation of water-soluble ammonia, may accumulate in the AP. Moreover, considerable concentrations of oxygen and phosphorus are also found in this fraction. The recirculation of AP in hydrothermal processes has gained significant interest, as it enhances biocrude production while reducing the costs associated with wastewater disposal. Several studies have demonstrated the positive impact of AP recirculation on biocrude and hydrochar yields, as well as its influence on microbial growth. In a comparative economic analysis of various AP treatment methods and biocrude upgrading systems following HTL of algae, it was found that direct recycling of AP to the algae farm was the most cost-effective option. This approach has the potential to reduce nutrient costs and improve overall sustainability [34,92,135,160,176,177,178,179,180,181,183,184,185,203]. 

Biomass cultivation using AP as a culture medium has been reported as an economically cost-effective route, leading to high rates of nutrient recovery. However, it should be noted that the high concentration of organic matter, including toxic heavy metals, aromatics, and nitrogen-containing compounds (e.g., 2-propenol, 2-propenal, aziridine, and 2-methylaziridine) in AP requires heavy dilution before microbial cultivation—thus leading to higher water consumption and increased operational costs [176]. In fermenting HTL-AP from *Spirulina*, microorganisms were somehow inhibited beyond 5% concentration and in full at 24%. Processing primary clarifier sludge revealed high organic pollution levels when treated with zeolite and iron-ammonium alum. The experimental use of copper and nickel sulfate as catalysts lowered organic compound content, with nickel sulfate causing the highest toxicity due to its toxic effect. All wastewater samples proved toxic to *Paramecium caudatum*, with nickel sulfate-treated samples being the most lethal, even at ten-fold dilution. HTL-AP from primary sludge was highly toxic to crustaceans, even at substantial dilution, emphasizing its inherent toxicity. However, a 1000-fold dilution formed samples without toxic effects on the test organisms in most cases. Aeration of high surfactant-concentration wastewater resulted in foam formation, removing sludge microorganisms. Cultivating activated sludge showed varying COD values, notably high for HTL-AP using nickel sulfate. Although most samples exhibited 70% efficiency, some catalyst-treated samples showed higher assimilation efficiency by activated sludge, indicating potential biodegradation [204]. Another study demonstrated the significant cytotoxicity of nitrogen organics extracted from HTL-AP, leading to a 50% reduction in cell density at a 7.5% raw HTL aqueous phase concentration [205]. Heavy metals like Pb, Zn, Cu, Cd, Cr, and Ni, present in HTL-AP, especially from sludge and manure sources, raise significant environmental and safety concerns. However, studies suggest that the HTL process can mitigate these risks by reducing the contamination levels of metals compared to raw feedstock [206].

The biocrude composed of heavy bio-oil/chemicals and light bio-oil/chemicals has been widely used for the production of various types of transportation fuel (e.g., gasoline, jet fuel, and diesel), as well as other essential bio-based chemicals (e.g., 5-hydroxymethylfurfural). Finally, the gas phase in HTL—constituting a relatively marginal fraction of the mass balance (<10%), has applications in fuel and chemical synthesis due to the presence of CO_2_ (>90%), CH_4_, and H_2_. This rich gas can also be used as feedstock for the cultivation of microalgae and gas-fermenting bacteria, while it may alternatively be recirculated back to the HTL reactor [9,11,120,124,169].

The integration of various treatment methods, such as anaerobic digestion (AD), bio-electrochemical systems (BESs), and advanced oxidation processes, with AP valorization, has demonstrated favorable results—including increased methane production, enhanced chemical oxygen demand (COD) removal, and improved valorization efficiency. Nevertheless, substantial challenges persist, and further research is required to optimize these technologies and fully assess their technological and economic feasibility. Life-cycle assessment tools can be instrumental in measuring the environmental impact of these processes and in guiding comprehensive approaches designed to address constraints associated with HTL product treatment and utilization. In conclusion, the valorization of HTL products represents a complex and essential aspect of hydrothermal processing, with enormous potential for sustainable and resource-efficient applications. Continued research and innovation in this field will undoubtedly pave the way for a greener and more sustainable future [6,19,32,169,182,185,186,189,207,208].

## 6. Economic Viability and Life-Cycle Assessment 

The conduction of a comprehensive economic assessment of emerging technologies remains pivotal in gauging their feasibility and competitiveness against conventional processes. Such evaluations serve as a cornerstone for designing suitable production capacities and determining the requisite for total capital investments. Processes like gasification, liquefaction, and pyrolysis share a common objective: the breakdown of large biomass molecules at high temperatures, employing enthalpy to disrupt their structures. Each method follows distinct pathways—pyrolysis and gasification rapidly vaporize components, yielding hydrocarbons, aromatics, and oxygenates, while liquefaction employs solvent properties under intense pressure and heat to depolymerize biomass, resulting in compound recombination. Operating conditions notably influence the product spectrum; gasification generates syngas and tar, pyrolysis produces condensed phases and gases, and liquefaction yields a high-yield liquid product alongside the solvent, all necessitating further refinement for viable fuel use. Syngas requires purification and compositional adjustment before catalytic conversion into fuel, whereas bio-oils undergo hydrotreatment to rectify undesirable properties. These processes vary in energy consumption, chemical replacement rates, emissions, and byproduct generation, impacting their economic viability and environmental implications. Despite approaching petroleum counterparts, their refined products maintain distinct chemical profiles, affecting refinery efficiency and suggesting alternative blending strategies for better composition homogeneity, particularly for distillation unit processing based on distillation curve analyses [7,29,209].

Estimates of the production costs of biofuels vary across different pathways; gasification stands at USD 1.6–5.50/GGE, fast pyrolysis-upgrading at USD 2.6 to 9.3/GGE, and HTL at USD 3.1–4.44/GGE for second generation feedstock (waste biomass and non-food crops). When using third-generation feedstock (algae), HTL emerges as more cost-effective, approximately USD 2.6/GGE, compared to gasification-FTS (USD 7.9/GGE) and pyrolysis (USD 8.1/GGE), mainly due to its capability to process high-moisture feedstock without drying costs. However, HTL remains relatively new without established pilot or demonstration-scale plants, leading to uncertainty in equipment cost estimates and plant design. The cost range of fermentation remains ambiguous due to limited available data. The disparity in production costs of thermochemical biofuel pathways, ranging from USD 1.93 to USD 7.11/GGE, stems from different economic assumptions and research scopes. Challenges in biofuel production include high feedstock costs, particularly with first-generation feedstock (food crops), and transportation difficulties due to low feedstock density. Feedstock costs, covering growing, harvesting, transportation, and pretreatment, comprise 40–60% of biofuel production expenses—and vary from USD 60–94/MT for woody and herbaceous energy crops to USD 15–50/MT for forest and agricultural residues. Therefore, the sustainability and efficiency of biofuel technologies heavily hinge on feedstock type and associated expenses [171].

Pyrolysis-derived bio-oil necessitates substantial upgrading due to its high oxygen content, low hydrocarbon yields, low energy density, and poor flowability. On the contrary, HTL, by heating the entire biomass, including water, potentially reduces external heating needs due to the contribution of water latent heat. Comparative assessments favor HTL over pyrolysis, demonstrating higher bio-oil yield, while bio-coal and non-condensable gases remain comparable in both technologies. Energy cost estimates highlight the advantages of HTL, with at least a 35% reduction in thermal energy costs compared to drying biomass. HTL emerges as a preferable technology in terms of energy product composition, yield, and efficiency—showcased by studies comparing biomass conversion such as *Chlorella* slurry, where fast pyrolysis required approximately 1.6 times more energy than HTL [210].

In another study, however, three plants comparing gasification (USD 68 million), liquefaction (USD 73 million), and pyrolysis (USD 52 million) in terms of total capital costs revealed negative net present values (NPVs) for all, with gasification showing the highest revenue and margin at USD 128.3 million NPV, followed by liquefaction (USD 113.7 million) and pyrolysis (USD 65.7 million). Sensitivity analysis highlighted the significant influence on NPV capital costs, especially in pyrolysis, thus emphasizing the financial risks of construction. Calculated minimum selling prices (MSPs) for profitability included gasification (USD 1.94/L), liquefaction (USD 0.98/L), and pyrolysis (USD 1.19/L). Operational breakdowns revealed feedstock costs as a significant factor, contributing 33% to pyrolysis and around 15% to gasification and liquefaction operating costs—with heating impacting gasification and ethanol replacement notably influencing liquefaction costs. Incentives to enhance process efficiency, manage feedstock expenses, and optimize cleaner production technologies are imperative by identifying areas for further research, like refining crude products to meet fuel standards and exploring stochastic methods for parameter modeling [209].

Techno-economic studies of HTL using woody biomass on a bench scale, sponsored by the National Advanced Biofuels Consortium (NABC), unfolded room for potential future improvements and highlighted the importance of reducing organic losses to the aqueous phase, thus directly impacting yields and wastewater treatment costs. Factors influencing bio-oil production costs encompassed feedstock expenses, product yields, and equipment upgrade costs. Studies focusing on lipid-extracted microalgae for liquid fuel production through HTL and upgrading processes indicated their potential as substitutes for conventional gasoline and diesel but emphasized sensitivity to uncertainties in feedstock costs—as well as the need for optimization, shorter residence time and alternative energy sources like CSP for heating the HTL reactor. Additionally, improved cultivation, harvesting, and dewatering methods were highlighted to reduce feedstock costs. The cost of hydrogen, essential in catalytic upgrading of bio-oil, emerged as a key consideration in HTL economics [94,116,117,118].

Economic models based on a biorefinery using HTL to produce biofuels from microalgae, with a daily capacity of 2000 dry tons, showed a calculated minimum fuel selling price (MFSP) of USD 679 per cubic meter [172]. Similarly, economic models of commercial-scale HTL facilities suggested competitive fuel prices ranging from USD 0.61 to USD 1.29 per liter of gasoline-equivalent [169]. Sensitivity analyses revealed that product fuel yield significantly impacted MFSP, stressing the importance of conversion achieved through HTL. Feedstock costs were identified as a critical factor influencing MFSP. Integrating hydrothermal treatments with anaerobic digestion (AD) for sewage sludge treatment offers environmental benefits and enhances the gross energy efficiency of AD, but requires further optimization to address associated economic concerns [3,116,117,118,172,201,202,208].

Comparative life-cycle assessments evaluating different HTL pathways for converting microalgae into biofuels considered parameters like energy efficiency, greenhouse gas emissions, energy return on investment (EROI), and net global warming potential (GWP). These assessments, along with detailed economic models for commercial HTL operations, pointed at competitive prices for biocrude, renewable diesel, and renewable jet fuels in most cases. Presently, commercializing hydrothermal treatment technology for wet waste faces challenges such as capital investment, processing times, and developing markets for final products. Despite these challenges, hydrothermal processing offers an appealing solution for wet waste management, converting waste into value-added products while eliminating biological contaminants [3,99,152,156,167,169,202].

An extensive study delved into the comparative evaluation of hydrothermal treatment (HTT), coupled with carbon capture and storage (CCS), against traditional bioenergy with carbon capture and sequestration (BECCS). Machine learning models were used to predict product yields and characteristics from HTT of diverse feedstocks, integrating these outcomes into an LCA model. Results highlighted the effectiveness of random forest models in predicting product yields and characteristics from HTT, especially for variable feedstocks. The HTT-CCS system showed potential as a net-energy-producing negative emissions technology (NET) for specific feedstock characteristics and reaction conditions, particularly demonstrating better EROI for lignocellulosic biomass at low temperatures. However, compared to conventional BECCS, HTT-CCS exhibited higher EROI but lower GWP—thus unfolding a tradeoff between energy yield and CO_2_ sequestration. Factors like feedstock properties significantly influenced the energy and environmental profiles of both systems, with wetter feedstocks requiring energy-intensive drying processes in the BECCS system that impact EROI and net GWP. The study suggests that decision-makers must prioritize either energy production or greenhouse gas sequestration when choosing between HTT-CCS and BECCS systems, as neither option stands out for both criteria. Additionally, the comparison with existing data suggests that ethanol production from sugarcane with CCS may represent the optimal BECCS performance for liquid fuel [211]. 

A thorough investigation explored the potential of HTL on food waste using a pilot-scale reactor and evaluated its viability as a commercialized renewable energy technology. The pilot-scale reactor outperformed its lab-scale counterparts, yielding higher biocrude oil (29.5 wt.% vs. 21.9 wt.%) at 300 °C and 60 min retention time. TEA estimated an MSP of biocrude oil at USD 3.48 per gallon gasoline-equivalent (GGE) in a base case scenario. Comparisons between on-site and mobile HTL reactor operations highlighted the latter’s profitability, especially when food waste sources were widely distributed (over 106 miles). When assessing economic feasibility among different feedstocks, food waste demonstrated better potential due to higher volatile compound content, yielding lower biocrude MSP at specific scales. However, at lower yields, this process became less economically feasible. Larger-scale HTL processes, particularly in urban areas with efficient waste-management networks, showed greater promise. Factors like natural gas prices, feedstock costs, and biocrude yield significantly influenced the selling price. Government policies supporting renewable biofuel technologies and higher biocrude yields were crucial for economic feasibility. Notably, mobile HTL scenarios proved advantageous in certain contexts, especially in rural areas with widespread food waste generation that facilitate more cost-effective operations. The choice of HTL system location, transportation methods, and local policy incentives heavily impacted its economic performance, emphasizing the importance of detailed site-specific analysis [212].

Such economic evaluations illuminate the viability and potential of HTL and related processes, emphasizing cost optimization, improved technologies, and innovative approaches as key drivers for the successful implementation and commercialization of these transformative biotechnological solutions.

## 7. Conclusions and Future Perspectives 

After years of extensive research, the application of hydrothermal processing to produce biocrude from non-food feedstocks has emerged as a promising approach to address the pressing challenges posed by fossil fuel depletion, global warming, and an ever-expanding global population. Biocrude derived from HTL can be further refined into a wide array of bio-based fuels and chemicals, making it a viable alternative to conventional petroleum-based products. 

In the pursuit of large-scale HTL operations, the availability of feedstock appears to be a critical consideration since transportation logistics can incur significant costs. Strategically separating the HTL plant from the upgrading unit, along with transporting biocrude to another location for upgrading, should be meticulously planned to optimize the process and minimize unnecessary expenses. HTL technology also offers an opportunity to address the mounting waste generation in urban areas by providing a sustainable and renewable source of energy and materials while simultaneously addressing waste treatment challenges. Nevertheless, several challenges persist in the HTL of biomass, including corrosion issues, energy and conversion efficiency, product separation, biocrude stabilization, water management, and process costs. The formation of char and coke during the process poses a considerable concern due to its adverse effects on biocrude yield and reactor blockage. Innovations in the development of catalysts, as well as continuous and high-pressure feeding systems, offer promising solutions to effectively overcome this issue, thus ensuring the economic feasibility and practicality of HTL. Recoverable and efficient catalysts play a crucial role in improving the quality of products, and thus the economic feasibility of the HTL process. Improving the physical and chemical properties of biocrude to resemble those of petroleum-based fuels is vital. Efficient and cost-effective upgrading processes, such as catalytic hydrotreatment, are focal points for further research.

By harnessing the potential of HTL process streams and integrating them into other applications, such as syngas production or wastewater treatment, the overall process efficiency and economics can be significantly improved. Looking forward, it can be concluded that circular economy studies, life-cycle analyses, and government support will be pivotal in advancing HTL technology toward commercial implementation. Moreover, gradual decentralization of hydrothermal waste treatment is anticipated in the long run. In this vision, both household and industrial waste would be handled in situ, leading to a reduction in environmental impacts, and further democratization of the energy sector. The application of efficient resource recovery principles would enhance the sustainability of this approach. By addressing technical barriers and exploring innovative market integration strategies, HTL products can transform the landscape of energy production and waste management and accordingly foster a more sustainable and circular economy. 

In conclusion, the remarkable progress in hydrothermal treatment of biomass, coupled with recent pilot plant and techno-economic assessments, demonstrates the immense potential of HTL technology. To fully realize this potential, continued research and collaboration across disciplines will be instrumental in making HTL a pivotal contributor to a greener and more sustainable future.

## Figures and Tables

**Table 1:** Main studies on the catalytic hydrothermal liquefaction of biomass.

Feedstock	Catalyst	Temperature (°C)	Time (min)	Effect on Bio-Oil Yield (%)	Reference
*Nannochloropsis* sp.	Pd/C	350	60	20	[75]
*Nannochloropsis* sp.	Pt/C	350	60	10	[75]
*Nannochloropsis* sp.	Ru/C	350	60	13	[75]
*Nannochloropsis* sp.	Ni/SiO_2_-Al_2_O_3_	350	60	10	[75]
*Nannochloropsis* sp.	MoCo/γ-Al_2_O_3_	350	60	15	[75]
*Nannochloropsis* sp.	Zeolite	350	60	8	[75]
*Nannochloropsis* sp.	Pt/C with H_2_	360	60	5	[75]
*Dunaliella tertiolecta*	5% Na_2_CO_3_	350	50	5.8	[76]
*Spirulina platensis*	Na_2_CO_3_	350	60	11.7	[77]
*Chlorella pyrenoidosa*	NaOH	240–280	30	10	[78]
*Chlorella pyrenoidosa*	Ce/HZSM-5	300	20	33	[79]
*Microcystic viridic*	Na_2_CO_3_	300–340	30–60	33	[80]
*Nannochloropsis* sp.	Nano-Si/SiO_2_	210	60	5.8	[81]
*Nannochloropsis* sp.	Nano-Si/SiO_3_	250	60	6.8	[81]
*Nannochloropsis* sp.	Nano-Si/SiO_4_	250	60	9.8	[81]
*Dunaliella tertiolecta*	Co/CNTs	320	30	9	[82]
*Dunaliella tertiolecta*	Ni/CNTs	320	30	9	[82]
*Dunaliella tertiolecta*	Pt/CNTs	320	30	5	[82]
Water hyacinth	0.5 N K_2_CO_3_	280	15	2	[83]
Water hyacinth	0.5 N KOH	280	15	3	[83]
Water hyacinth	1 N K_2_CO_3_	280	15	6	[83]
Water hyacinth	1 N KOH	280	15	7	[83]
*Dunaliella tertiolecta*	5 wt.% Na_2_CO_3_	300	60	7.7	[84]
*Microcystic viridic*	5 wt.% Na_2_CO_3_	340	30	7.5	[80]
*Enteromorpha prolifera*	5 wt.% Na_2_CO_3_	300	30	2.6	[85]
*Spirulina platensis*	5 wt.% Na_2_CO_3_	350	60	11.7	[77]
*Chlorella pyrenoidosa*	5 wt.% Na_2_CO_3_	280	30	5	[78]
*Nannochloropsis* sp.	Na_2_CO_3_	210	60	1.7	[81]
*Nannochloropsis* sp.	Na_2_CO_3_	230	60	3.8	[81]
*Nannochloropsis* sp.	Na_2_CO_3_	250	60	4	[81]
Corn stalk	1 wt.% Na_2_CO_3_	300	30	13.8	[86]
Wood biomass	0.94 M K_2_CO_3_	280	15	25.2	[87]
*Dunaliella tertiolecta*	5% Na_2_CO_3_	360	50	25.8	[76]
Pretreated sorghum bagasse	Formic acid	300	60	17	[88]
Pretreated sorghum bagasse	K_2_CO_3_	300	60	39	[88]
Pretreated sorghum bagasse	KOH	300	60	18	[88]
Pretreated sorghum bagasse	Formic acid	350	60	31	[88]
Pretreated sorghum bagasse	K_2_CO_3_	350	60	30	[88]
Pretreated sorghum bagasse	KOH	350	60	29	[88]
Barely straw	K_2_CO_3_	300	30	14	[50]
*Cladophora glomerata*	3D composite of hydrochar, zeolite, and magnetite	320	20	-	[89]
*Cladophora glomerata*	graphene oxide/polyurethane composite	320	20	54	[55]
*Spirulina* sp.	Ni/Reduced graphene oxide	270	30	9	[54]
*Prosopis juliflora* (hardwood waste) and polypropylene	Nb/Al_2_O_3_	420	60	22.6	[61]
*Spirulina platensis*	Ni/Biochar	350	34	6.4	[53]

**Table 2:** Key Studies and research contributions in the field of HTL.

Plant/Institution/Company	Reactor Type	Feedstock	Temperature (°C)	Pressure (bar)	Residence Time (min)	Catalyst	Throughput (kg/h)	Reference
Albany, NY, USA (PDU–PERC, PDU-LBL)	Tubular/stirred	Wood	330–345	207	11–465	Na_2_CO_3_	43–360	[120]
Berkeley, CA, USA (LBL)	Stirred	Wood	330–350	200–230	20	-	1	[121]
U.S. Environmental Protection Agency, Washington, DC, USA, (STORS–EPA)	Column	Sewage sludge	275–305	86–148	90	Na_2_CO_3_	30	[122]
Organo Corp., Tokyo, Japan (STORS)	Column	Sewage sludge	290–300	88–98	-	-	240	[123]
Shell, Amsterdam, The Netherlands (HTU^®^)	Tubular	Wood	350	180	6	-	10	[124]
Biofuels B.V., Amsterdam, The Netherlands (HTU^®^)	Tubular	Sugar beet pulp and onion pulp	350	180	15	-	10	[125]
Pacific Northwest National Laboratories, Richland, WA, USA	Stirred + tubular	Algae, macroalgae, grape pomace, and wastewater solids	350	200	27–50	Na_2_CO_3_	1.5	[45,46,126,127,128]
University of Sydney, Australia	Coils in sandbath	Algae	350	200	15–20	-	24–40	[67,104]
University of Illinois, USA	Stirred	Swine manure	350	103	40–80	-	0.9–2.0	[35,129]
Iowa State University, USA	Tubular	Fungi	300–400	270	11–31	-	3.0–7.5	[130]
Chalmers University of Technology, Sweden	Fixed bed with recycle loop	Kraft lignin	350	250	6–11	ZrO_2_ and K_2_CO_3_	1–2	[33,37,38,48]
Aalborg University, Denmark	Tubular	Wood/glycerol	390–420	300–350	15	K_2_CO_3_	20	[71,131]
Karlsruhe Institute of Technology (KIT), Germany	Tubular/stirred with recirculation or with MeOH gasifier	Waste biomass (algae, yeast, or pomace)	330–450	200–250	1–30	K_2_CO_3_ and ZrO_2_	0.06–0.63	[42,132,133]
University of Leeds, UK	Coils in sandbath	*Chlorella*	350	185	1.4–5.8	-	0.6–2.4	[134]
Aarhus University, Denmark	Tubular with oscillator	Wood, sewage sludge, and *Spirulina*	350	220	10	KOH	60	[105,107,135]
Imperial College London, UK	Tubular	Algae	300–380	180	0.5–4	Hexane	0.03–0.24	[91]
Bath University, UK	Concentric tubular	Wastewater and algae	302–344	160	17.7–41.8	-	0.18–0.42	[106]
University of Twente, The Netherlands	Coils in sandbath	*Scenedesmus*	250–350	150–300	7–30	-	0.06–0.33	[41]
Steeper Energy, Vedbæk, Denmark, Calgary, AB, Canada (Hydrofaction^™^)	Tubular	Wood	390–420	300–350	15	K_2_CO_3_	20	[136,137]
Muradel, Whyalla, Australia (Green2black^™^)	Tubular	Tires and algae	360	200	10	-	168	[102]
Genifuel, Salt Lake, UT, USA (HTP)	Tubular/stirred	Sewage sludge	350	150–300	45	-	200	[102]
ENI S.p.A., Milan, Italy (W2F)	-	Organic fraction of municipal solid waste	250–310	100	60–120	-	1–5	[102]
SCF Technologies, Herlev, Denmark (CatLiq^®^)	Stirred	Wet digested grains with solubles	350	250	1–15	ZrO_2_	30	[138]
Altaca Enerji, Istanbul, Turkey (CatLiq^®^)	-	Different wastes and residues	250–350	150–300	7–30	-	15,000	[139]
Changing World Technologies, West Hempstead, NY, USA (TDP process)	-	Turkey waste	200–300	-	-	-	8500	[102]
Institute of Nuclear and New Energy Technology, Tsinghua University, China	Tubular	Coal and microalgae	340	250	30	Ethanol	0.05–0.1	[140,141]
Indian Institute of Technology, India	Stirred	Macroalgae, hardwood black liquor, rice straw, algae-treated dairy wastewater, microalgae-bacteria consortium, municipal solid wastes	300–350	220–250	30–55	KOH, Na_2_CO_3_, and glycerol	0.03–0.05	[70,142,143,144,145,146,147,148]
Korea Institute of Science and Technology, Korea	Stirred	Lignin	300–350	200	28–300	Ethanol	5	[149]

**Table 3:** Applications of HTL products.

Phase	Applications	Examples/Specific Uses	References
Liquid (hydrophobic)	Renewable energy generation, transportation fuel, chemical feedstock, biorefinery supply, electricity generation, residential heating, energy storage, bio-based products	Gasoline, jet fuel, diesel, power generation	[1,7,8,9,11,13,21,23,24,29,43,88,93,94,117,118,120,124,128,132,134,136,150,153,164,167,169,170,171,172]
Liquid (hydrophilic)	Nutrient recovery, biogas production, wastewater treatment, biochemical and biomaterial supply, algal biomass recovery, carbon sequestration	Microbial fuel cell (MFC), anaerobic digestion, microbial electrolysis cell (MEC), biobatteries, algal cultivation, fertilizer production, power generation, HTL recirculation	[34,92,135,144,173,174,175,176,177,178,179,180,181,182,183,184,185,186]
Solid	Soil amendment, carbon sequestration, waste minimization and valorization, adsorbent material, energy generation, biogas production	Wastewater treatment, soil amendment, nanomaterial manufacture (e.g., graphene), fertilizer production, biofuel, catalyst manufacture, power generation, HTL recirculation	[53,182,187,188,189,190,191,192,193,194,195]
Gas	Renewable energy generation, biogas production, hydrogen production, chemical synthesis	Biofuel, fermentation, algal cultivation, hydrogen source, HTL recirculation	[7,93,117,196,197]

## Data Availability

Not applicable.
